# Role of Memantine in Limiting Cochleotoxicity in Rats

**DOI:** 10.1007/s12070-024-04521-1

**Published:** 2024-02-16

**Authors:** Pavlos Pavlidis, Vasilis Spyridon Tseriotis, Kyriaki Papadopoulou, Sophia Karachrysafi, Chrysanthi Sardeli, Haralampos Gouveris, Faye Malliou, Dimitrios Kavvadas, Theodora Papamitsou, Antonia Sioga, Penelope Anastasiadou, Dimitrios Kouvelas

**Affiliations:** 1 Department of Otorhinolarhingology / Head & Neck Surgery, University Medical Center Mainz, Mainz, Germany; 2 Laboratory for Clinical Pharmacology, School of Medicine, Faculty of Health Sciences, Aristotle University Thessaloniki, Thessaloniki, Greece; 3 Laboratory of Histology-Embryology, School of Medicine, Faculty of Health Sciences, Aristotle University of Thessaloniki, Thessaloniki, Greece; 4 Department of Oral Medicine/Pathology, School of Dentistry, Faculty of Health Sciences, Aristotle University of Thessaloniki, Thessaloniki, Greece

**Keywords:** Memantine, Amikacin, DPOAEs, Cochleotoxicity, Memantine

## Abstract

Οur aim was to test whether amikacin’s well-known cochleotoxic effects could be suppressed, depending on whether an NMDA-antagonist (memantine) was administered simultaneously with or after amikacin treatment. Forty Wistar rats were used in this experiment. Ten rats acted as controls and received no medication (group A). Amikacin (200 mg/kg) was administered intraperitoneally (i.p.) once daily for 14 days to 10 animals in group B; amikacin (200 mg/kg) was administered concurrently with memantine (10 mg/kg, i.p., once daily) to the same 10 animals in group C. Group D was given intraperitoneal memantine (10 mg/kg, once daily) for 14 days following a 2-week amikacin treatment. The cochlear activity of the right ear was tested using DPOAE in conscious animals. All animals were sacrificed at the conclusion of the experiment and both cochleae were collected for histological and immunohistochemical analysis. All groups treated with amikacin showed decreased cochlear activity, as testified by decreased DPOAE-amplitudes compared to the pre-treatment state. In the rats of group B, the DPOAE reduction was more pronounced. On histologic exam, the cochlear structures of group C rats and, although to a lesser extent, group D rats showed less severe cochlea damage. Memantine plays a protective role, resulting in restoring partially cochlear structures when administered either simultaneously with or after completion of amikacin i.p. treatment in rats.

## Introduction

Aminoglycoside antibiotics have been in clinical use for over 50 years and presently are of growing importance [1–3]. No matter their advantageous therapeutic attributes, using these antibiotics is limited due to their significant ototoxicity that can result in permanent hearing loss [1, 4]. Cochlear outer hair cells are mainly prone to aminoglycosides’ toxicity, and the loss of these cells possibly represents the cellular foundation for aminoglycoside-associated hearing loss [1]. The excitatory amino acid glutamate is the most crucial afferent neurotransmitter within the peripheral auditory system device [3]. Glutamate is released from the inner hair cells and binds to N-methyl-D-aspartate (NMDA) receptors placed on terminals of spiral ganglion neurons. NMDA receptors are expressed inside the cytoplasm of the auditory neurons, and very low concentrations of NMDA receptor subunits’ mRNA have been detected in other regions of the cochlea [1, 5].

Although the mechanism responsible for aminoglycoside-associated hearing loss is not yet fully elucidated, NMDA receptors are strong candidate molecules [6–9]. Evidence suggests that aminoglycosides can mimic the modulatory actions of polyamines on the NMDA receptor, causing excitotoxicity and cell death [9–12]. Recently, it was shown that aminoglycoside-induced hair cell loss could be significantly reduced in the presence of NMDA antagonists [13, 14].

In the present study, we investigated the functional disorders and morphological changes of the cochlea in rats after intraperitoneal (i.p.) administration of amikacin. In addition, we investigated the possible protective action of memantine, an established NMDA-antagonist, when administered either concurrently with or after the end of a course of aminoglycoside treatment.

## Materials and Methods

### Animals

In total, 40 male Wistar rats with documented dates of birth (8 weeks old) were obtained through the Vivarium of the Veterinary School of our University and included in the study. All rats were housed with free access to food and water. No outer or middle ear pathologies were observed. The animals were divided into 4 groups (10 rats in every group). The first group was used as the control group (**group A**). The second group was injected daily with a low dose of amikacin (200 mg/kg, Sigma-Aldrich, USA, once per day) and administered intraperitoneally (i.p.) for 14 consecutive days (**group B**). An additional group (**group C**) received amikacin i.p. with the same dosing schedule as group B and memantine (10 mg/kg/d, once per day, i.p, Sigma Aldrich, USA) in parallel. An additional 10 rats, (**group D**) received amikacin i.p. (200 mg/kg, once per day) for 14 days and then memantine (10 mg/kg/d, once per day,i.p) for another 14 days. It should be mentioned that there was no gap between stopping amikacin and starting memantine. It should be also made clear, that there was no specific rationale for treating these rats with memantine for 14 days. The researchers wanted only to administer both substances for the same time period.

### Distortion-Product Otoacoustic Emissions (DPOAEs)

In conscious animals, the cochlear activity of the right ear of all rats was examined thrice during the experiment using DPOAEs (day 0 = just before the administration of the first amikacin dose, day 7, and day 14). The Vanderbilt protocol for DPOAE measurements was used. The DPOAE test protocol included an f2/f1 ratio of 1.22, stimulus intensity levels of L1 = L2 = 65 dB SPL, f2 values from 593 to 6781 Hz (3 test frequencies/octave), and 16 averages/frequency .

Two additional DPOAE measurements were obtained on days 7 and 14, after the discontinuation of amikacin to detect any possible delayed deterioration or improvement of the cochlear activity (groups B and C). The same procedure was applied to the rats in group D.

The DPOAEs at 2f1/f2 were elicited from the control and experimental animals utilizing an ILO-96 cochlear emission analyzer (Otodynamics, London, UK). The intensities of primary stimuli were set at the same level (L1 = L2), namely at 65 dB SPL. The frequencies (f1 and f2) were adjusted as f2/f1 = 1.21. The f2 frequencies examined for DPOAE-grams ranged from 1 to 6.3 kHz (1001, 1184, 1416, 1685, 2002, 2380, 2832, 3369, 4004, 4761, 5652, 6299 Hz). The primary tones produced by two separate speakers were introduced into the animal’s outer ear canal through an insert earphone probe. Detection threshold and suprathreshold measures in input/output (I/O) functions were obtained by decreasing the primary tones from 75 to 36 dB SPL in 3-dB steps. The DPOAEs were measured and recorded as an average of four separate spectral averages of each stimulus condition. The noise floor level was measured at a frequency that was 50 Hz above the DPOAE frequency, using similar averaging techniques [15].

### Preparation for Light Microscopy and Immunohistochemistry

After completion of the experiment, the animals were sacrificed and the cochlea was dissected into 0.5- to 4.0-cm-thick tissue blocks and fixed by immersion in a 10% formalin solution (from a 35% formaldehyde stock solution). Samples were then decalcified and dehydrated through an ascending series of alcohol solutions (76%, 96%, 100%, 100%). The tissues were then cleaned in xylene for four hours. They were then immersed in liquid paraffin for an additional four hours to allow embedding. For this purpose, the samples were placed in metal molds, immersed in liquid paraffin and cooled at 4 °C for twenty minutes [16].

The paraffin blocks were then sectioned using a semi-automatic microtome at a thickness of 3 μm. Ten sagittal sections were taken at random. Two of these sections were placed on standard slides, while the other eight were placed on seven positively charged slides. They were all dried at room temperature for one hour. The first two sections, intended for morphological analysis, were placed in the oven at 65 °C for one hour and then immersed in xylene solution for ten minutes. They were then hydrated with a descending series of alcohol solutions (100%, 100%, 96%, 76%). The sections were then stained with hematoxylin for five minutes and rinsed in tap water for another five minutes. For partial staining with hematoxylin, 1% differentiation solution was used for one second. Sections were stained with eosin for one minute, dehydrated in ethanol for five minutes, and cleared in xylene for another five minutes. Finally, the slides were covered with “Canada balsam” for light microscopic analysis [16].

The other eight positively charged slides were used for immunohistochemical analysis using anti-TGF-β1 [Santa Cruz, USA, dilution 1:50] and anti IL -6 [Santa Cruz, dilution 1:100] antibodies. The intensity of staining was assessed by semiquantitative evaluation based on a cross-count scale according to the following categories: negative – no cross (-), mild – one cross (+), moderate – two crosses (++), strong – three crosses (+++). All slides were examined under bright field microscope by at least two independent observers blinded to the identity of immunohistochemical preparations. For all slides, special attention was paid to the scala vestibuli, scala media, and scala tympani, as well as to the basilar membrane (BM), organ of Corti (OoC), Reissner’s membrane (RM), spiral ganglion neurons (SGN), spiral ligament (SL), stria vascularis (SV), and tectorial membrane (TM).

Quantitative analysis of hair cells at each level of the basilar papilla was performed by viewing each section under an oil immersion objective (X40) at a total magnification of X500. To ensure consistency and reliability of hair cell counting, similar counting criteria as before were used: Presence of a well-formed cell body, extension of the cell to the cuticular plate, and detectable stereocilia. Three 4-µ sections were analyzed at each 100-µ interval, and the average number of hair cells per section was recorded. Counts were expressed as a function of distance from base and then normalized for all animals by converting to percent of total optic nerve length in 5% increments.

#### Counting of Hair Cells and Nuclei

As described in a previous study [17], hair cell counting was performed by an individual unfamiliar with the experimental procedure. Inner and outer hair cells were counted using Nomarski interference contrast (NIC) microscopy on a Nikon Optihot-2 microscope. The presence of a hair cell was defined as an intact, spherical nucleus in the basal half of the cell [17].

For basilar membrane reconstruction, low magnification images of serial cochlear slices were acquired using a QImaging Exi Aqua camera in combination with QImaging software (QImaging, Surry, British Columbia, Canada). These images were further analyzed using Adobe Photoshop, and the (x,y) coordinates of each basilar membrane point containing hair cells were identified and exported as a Microsoft Excel file. The basilar membrane coordinates were matched with the corresponding hair cell count data in Excel and ordered from the most basal point to the tip of the cochlea. The Greenwood equation was used to convert the relative location of the cochlea, expressed as a percentage from the apex (100% is the basal, 0% is the apex), into a characteristic frequency for data analysis [17].

Two different individuals performed the HC counts for the entire set of fully assembled dissected tissue. These two sets of HC counts were used to calculate inter-rater reliability. These two individuals focused on the nuclei of the HC and tried not to confuse them with the nuclei of the nearby supporting cells. **Spiral ganglion cells (SGC)**: The Rosenthal canal was divided into 4 segments as previously described [18] Segment I (from base to 6 mm), II (from 6 to 15 mm), III (from 15 to 22 mm), and IV (from 22 mm to apex). In each segment, all nuclei were determined by multiplying the total number by 10 and by a factor of 0.9 to account for cells that would be counted based on their location at the interface between sections. The density of SGC was determined as the number of nuclei per 10,000 µm2 Rosenthal canal on the middle turn [19].

#### Stria vascularis (SV)

The SV includes three cell types: marginal, intermediate, and basal cells [19]. Morphometric measurements of the area of the stria vascularis were also performed in all cochlear convolutions at the middle modiolar level and in the two adjacent sections. As described in a previous study, we acquired each image with a digital camera at a magnification of 200×. The secondary changes, such as cyst-like structure The secondary changes, such as cystic-like structural regions or concretions were excluded [17].

#### Spiral Ligament (SL)

The spiral ligament has been divided into 4 segments according to the appearance of different types of fibrocytes, based on previous studies [17, 20].

Type I fibrocytes lie circumferentially aligned between the stria vascularis and the bone. Type II fibrocytes occupy the superficial inferior spiral ligament between the basilar crest and the stria. Type I and II fibrocytes are important for potassium recycling [20, 21]. Type III fibrocytes are longitudinally located in the deepest part of the inferior spiral ligament. Type IV fibrocytes lie radially oriented, inferior to the basilar crest.

To evaluate the mean loss of fibrocytes in each segment, we used the following rating scale, as suggested previous studies [17]: 0, within normal limits (missing less than one-third of the fibrocytes); 1, missing one-third of the fibrocytes; 2, missing two-thirds of the fibrocytes; and 3, severe or complete loss of the fibrocytes on sections at the midmodiolar level. A magnification of x 40 has been applied.

### Statistical Analysis

We compared DPOAE amplitudes of each experimental group separately at the 1st, 2nd or 3rd measurement.

First, the mean DPOAE amplitudes were calculated for each specific frequency. Then, for each specific group we calculated the average DPOAEs of all frequencies. We used a one-way ANOVA or Kruskal-Wallis test, followed by post hoc tests or pairwise comparisons in order to compare the groups at each specific measurement. Moreover, a repeated-measures ANOVA with post-hoc tests was also used at that point, for comparisons of the average DPOAEs for each one of the experimental groups in different measurements.

Moreover, we conducted a second analysis, in which the average DPOAEs of different groups were compared at different frequencies of each measurement. The 1st, 2nd and 3rd measurement comprised DPOAE values recorded at the end of each respective week of the experimental procedure. The 4th measurement comprised values recorded at the end of the 3rd week for groups A, B, C and values recorded at the end of the 4th week of the experiment, since it was predetermined for group D to start memantine treatment at the end of the 2nd week and complete it 2 weeks afterwards.

The statistical programme R (Version 4.1.2) was used for all statistical analyses.

## Results

### Average DPOAEs Analysis

DPOAE amplitudes were used for the investigation of differences in cochlear activity between groups. At the 1st measurement (week 1) no significant results were noted between the four groups. At the 2nd measurement, DPOAEs recorded from control rats (group A) were characteristic of intact cochleae, whereas DPOAES from animals treated with either amikacin as monotherapy or amikacin in combination with memantine were indistinguishable from background noise, indicating severe loss of outer hair cell function. Of note, average DPOAE levels from groups B, C or D did not approach those of control rats in week 2, demonstrating significant difference (*p* < 0.001). No significant difference was found between the average DPOAEs of groups B, C or D. At the 3rd and 4th measurement, similar results were observed. Groups B, C and D differed significantly from the Control group (*p* < 0.001).

**Fig. 1:**
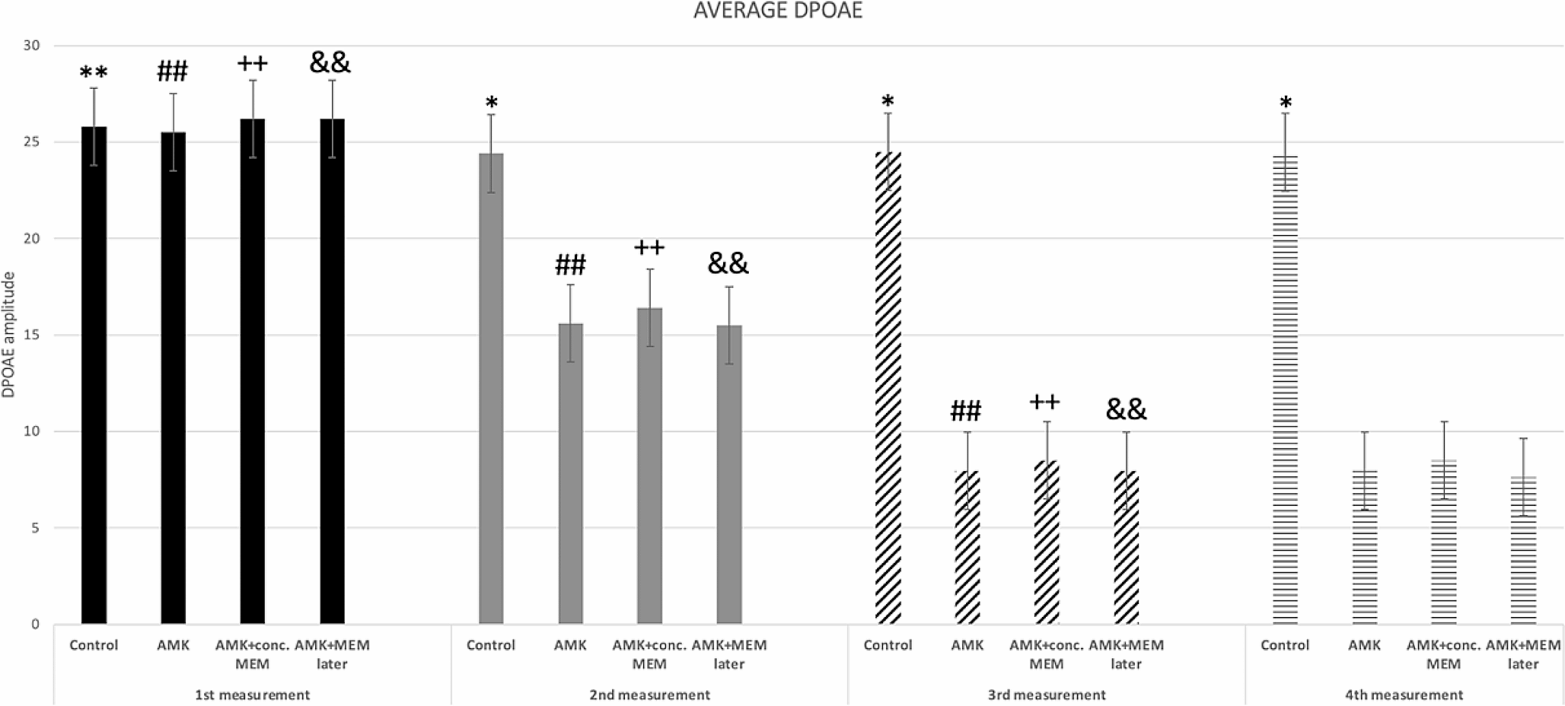
Average DPOAE amplitudes from all frequencies, categorized by group. * denotes significant difference (*p* < 0.001) between the control group and each one of the groups B, C, D at measurements 2, 3 and 4, while no difference was found between groups B, C, or D. One-way ANOVA with Tukey post-hoc tests. ** denotes significant difference (*p* = 0.006) of the initial measurement of the control group in comparison to the next measurements. ## denotes significant decrease of DPOAEs in group B at every next measurement (*p* < 0.001). Repeated-measures ANOVA with post-hoc tests. ++ denotes significant decrease of DPOAEs in group C at every next measurement (*p* < 0.001). Repeated-measures ANOVA with post-hoc tests. && denotes significant decrease of DPOAEs in group C at every next measurement until week 3 of the experiment (*p* < 0.001). Repeated-measures ANOVA with post-hoc tests

Figure 1 presents a bar plot of the groups’ average DPOAEs at different time points.

The analysis with a repeated-measures ANOVA unexpectedly showed significant differences in the average DPOAEs of the control group at the different time points. DPOAE amplitudes at the 1st measurement were significantly higher than the ones recorded at measurements 2 and 3 (*p* = 0.006). DPOAE amplitudes in groups B and C were significantly lower from previous records at every next measurement (*p* < 0.001), reflecting the damage in the outer hair cells. The same applied from the repeated measurements of group D (*p* < 0.001), without however any statistical difference between the 3rd and 4th week of the experiment (*p* = 0.45).

### Frequency-level DPOAEs Analysis

**Fig. 2:**
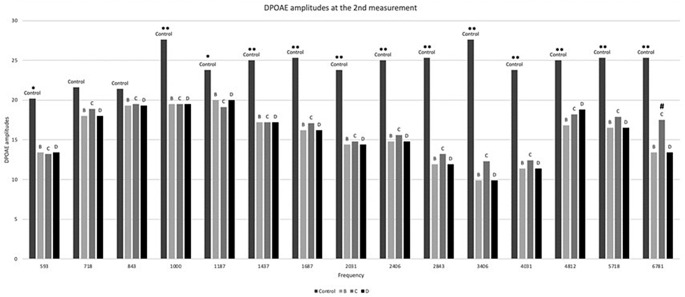
DPOAE amplitudes in week 2. * denotes significant difference (*p* < 0.05) between the control group and group C. ** denotes significant difference (*p* < 0.05) between the control group and each one of the groups B, C, D. # denotes significant difference (*p* < 0.05) between group C and either group B or D in frequency 6781 particularly. One-way ANOVA with Tukey post-hoc tests

DPOAEs from various frequencies did not differ significantly between any of the experimental groups in week 1, with results similar to the ones of the average DPOAE analysis described above. In week 2 groups B, C and D statistically differed from the control group (*p* < 0.05) at almost all frequencies, with the results being in concordance to the average DPOAE analysis. Interestingly, group C, that received both amikacin and memantine simultaneously, was the only group to have significantly lower DPOAE values compared to control group at 593 (*p* < 0.05) and 1187 Hz (*p* < 0.01). On the other hand, group C demonstrated less deterioration of cochlear activity compared to groups B and D at the highest frequencies, namely 6781 Hz (*p* = 0.002) (Fig. 2).

**Fig. 3:**
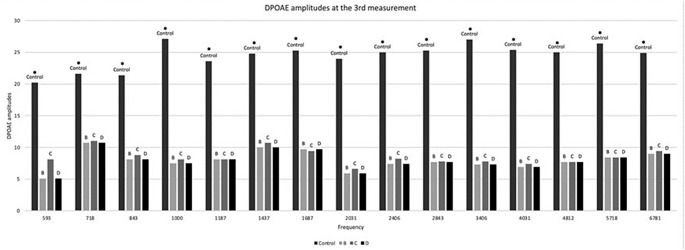
DPOAE amplitudes in week 3. * denotes significant difference (*p* < 0.05) between the control group and each one of the groups B, C, D. One-way ANOVA with Tukey post-hoc tests

**Fig. 4:**
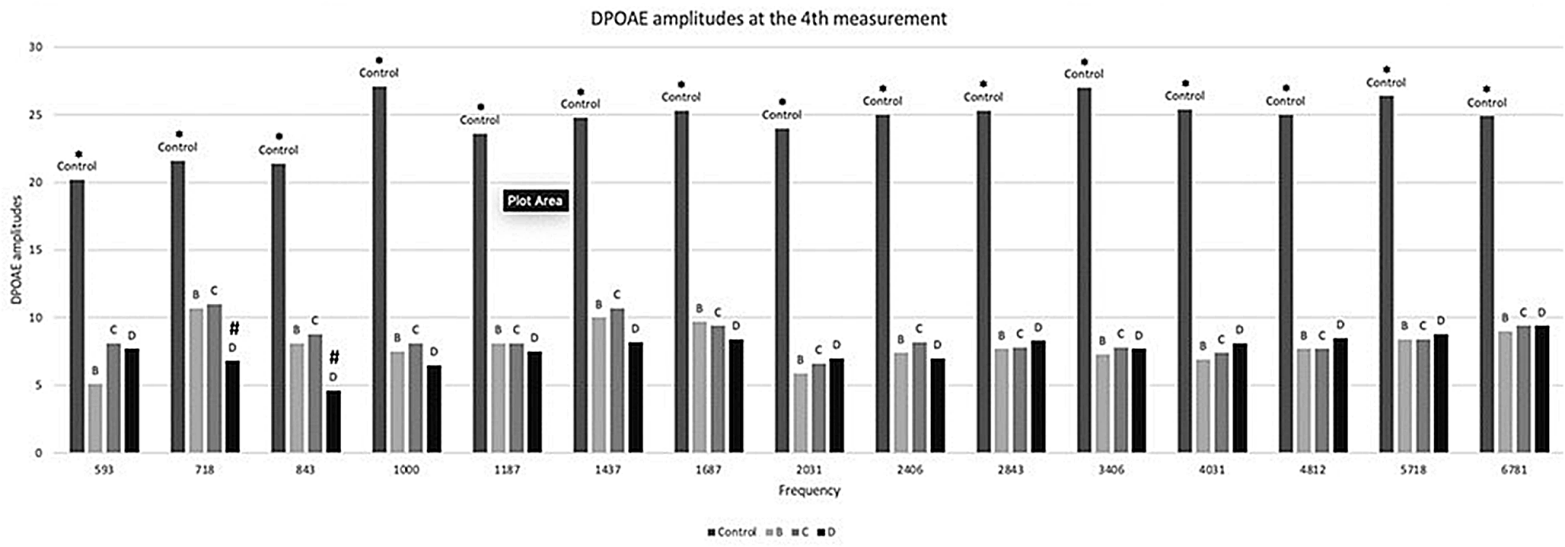
* denotes significant difference (*p* < 0.05) between the control group and each one of the groups B, C, D. # denotes significant difference (*p* < 0.05) between group D and either group B or C. Kruskal-Wallis non-parametric test with pairwise comparisons

In the 3rd and 4th measurement, significantly lower DPOAEs were found in groups B, C and D at all frequencies when compared to the control group (*p* < 0.01) (Figs. 3 and 4). In the 4th measurement, however, group D (in which memantine treatment started later) had significantly worse DPOAEs than groups B and C at the low frequencies, namely 718 and 843 Hz (*p* < 0.05).

### Histological Examination

Before evaluating hair cell counts as a function of acoustic exposure or tissue preparation, we performed a series of within-class correlation analyses to determine the reliability of HC count data. An analysis of “absolute agreement” ,” as opposed to “consistency”.” Blinded HC counts from two independent raters, using phalloidin-stained prepared tissue, were compared using an ICC analysis based on a mixed two-way model as proposed by Neal et al. [21]. When the ICC coefficients of rater 1 and rater 2 were compared for whole-body preparation, it was found that the two counts were in good agreement with respect to HC type or treatment conditions, with all ICC coefficients exceeding 0.9.

**Fig. 5:**
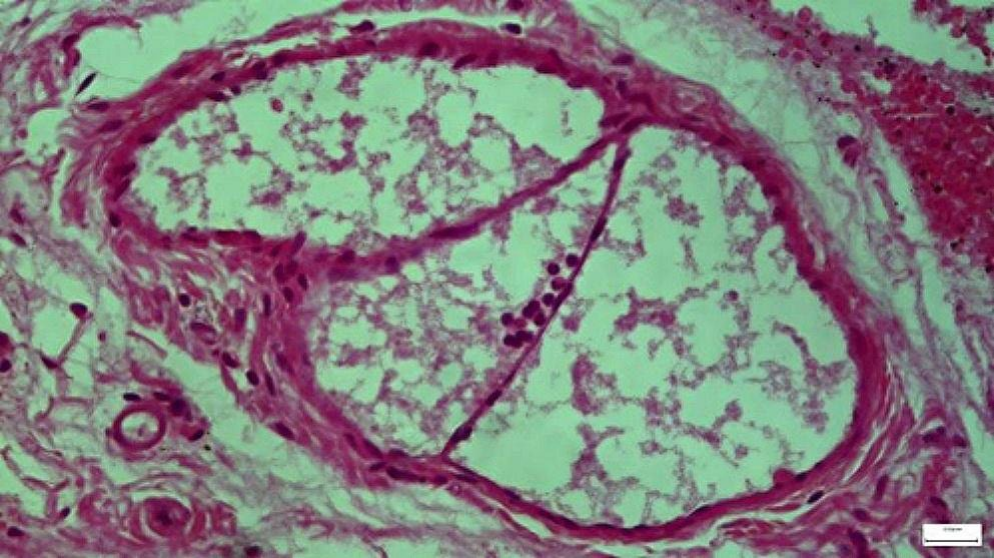
Rat specimen from Group A. Reissner’s membrane has no histological alterations. Increased eosin-hematoxylin staining

**Fig. 6:**
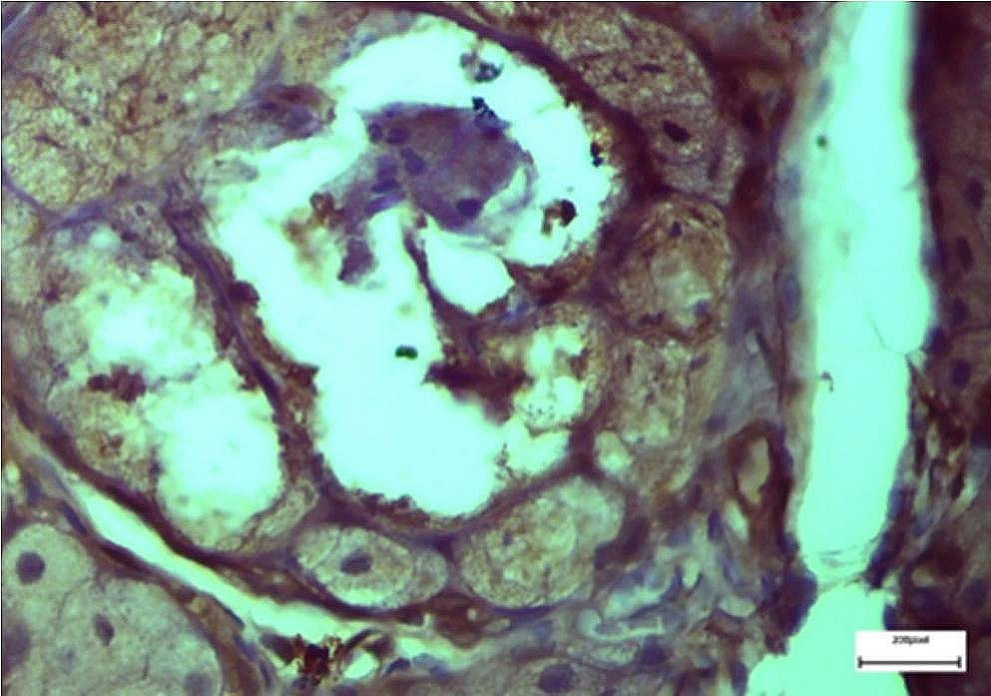
Rat specimen from Group B. Reissner’s membrane separation and cell flattening. Additionally visible is intense Eosin-hematoxylin staining

Light microscopic examination of the sections stained with eosin-hematoxylin in group A revealed no histopathologic changes in the Reissner membrane of the scala vestibuli, the scala media, and the scala tympani, as well as the basilar membrane (BM), the organ of Corti (OoC), the spiral ganglion neurons (SGN), the spiral ligament (SL), the stria vascularis (SV), and the tectorial membrane (TM, Fig. 5), whereas immunohistochemical staining for TGF-β1 and IL -6 markers were negative (-) in all specimens examined. In group B, light microscopic examination of sections stained with eosin-hematoxylin showed detachment of the vestibular (Reissner) membrane of the cells with flattening (Fig. 6). All samples showed immunohistochemical staining for the markers IL -6 and TGF-β1 as intense (+++). In group C, eosin-hematoxylin staining showed no pathological changes in the integrity and architecture of the vestibular (Reissner’s) membrane but a slight flattening of the cells. Immunohistochemical staining for IL -6 was detected as negative (-) in all samples. With regard to TGF-β1, the preliminary results suggest that TGF-β1-mediated apoptosis is not a possible mechanism of the observed lesions. In group D, eosin-hematoxylin staining showed detachment of the vestibular membrane (Reissner membrane) with slight shrinkage of the cytoplasm of the cells.

For this analysis we have also used a one-way ANOVA or Kruskal-Wallis test, followed by post hoc tests or pairwise comparisons. We found a significant difference in the mean (SD) total number of spiral ganglion cells between group A and groups B, C, and D (*P* = 0.001). We also found a significant difference in the mean (SD) number of spiral ganglion cells between groups B and C (*P* = 0.02) and groups C and D (*P* = 0.04). The same difference was found when the number of outer hair cells was counted. Interestingly, the inner hair cells were affected to the same extent in groups B, C, and D. The same statistical difference (*p* = 0.03) was found when the number of inner hair cells of groups B, C, and D were compared with that of group A.

We found a significant difference between group A and group B in the mean (SD) degree of atrophy of the stria vascularis (*p* = 0.01), between group A and group C (*p* = 0.03), and group A and group D (*p* = 0.02). The findings were the same regardless of which part of the stria vascularis we were comparing. Comparison between rats in groups B and C showed a significant difference between them (*p* = 0.02). A significant statistical difference was also found between groups B and D. This is indicative of the protective role of memantine when the latter is administered concomitantly with amikacin (*p* = 0.04). Interestingly, the protective role of memantine is lower when it is administered after treatment with amikacin (comparison between groups B and D, (*p* = 0.03).

At this point we would like to mention that once the *p* value has crossed the predetermined value of 0.05, the absolute value should not be used to declare “more significant” or “less” significant. Since the sample size of each group is rather small, it would be rather difficult to comment om “statistical” sup[eriority of one team over other.

## Discussion

Previous studies suggested that ototoxicity induced by aminoglycoside antibiotics is associated with excitotoxic activation of NMDA receptors in the cochlea. Our results suggest that memantine, when coadministered with amikacin, may reduce the cochleotoxic effects induced by amikacin. A smaller but nonnegligible reduction in efficacy was observed when memantine was administered directly after the cesation of the treatment with amikacin. The same functional results were observed with DPOAE. These results provide compelling evidence that activation of NMDA receptors on hair cells or their afferent synapses is a necessary step in aminoglycoside-induced hearing loss [22].

NMDA receptors are located at each synapse at the bottom of the mammalian auditory pathway. Their developmental regulation and unique inner ear subunit composition promote protective and neurotrophic functions after acute injury by regulating AMPA-R expression and helping to restore synaptic inputs. This is in contrast to chronic injury, which is associated with overactivation of NMDA-R and leads to nerve death. In our case, they are located at synaptic sites between cochlear hair cells and radical dendrites of spiral ganglia afferents. Aminoglycosides can mimic the action of polyamines on NMDA receptors [23]. The link between aminoglycosides and polyamines may explain the glutamate-like excitotoxicity induced by aminoglycosides. Overstimulation of NMDA receptors (NMDARs) increases the formation of nitric oxide (NO) and causes oxidative stress in hair cells. In addition, several studies have shown that gentamicin treatment can increase the expression of nNOS and iNOS and cause hair cell damage [24, 25].

There is evidence that aminoglycoside-induced cochleotoxicity results from excitotoxic processes mediated by the polyamine-like effects of aminoglycosides on NMDA receptors in the cochlea [25, 26]. Since the polyamine-like agonist properties of aminoglycosides should induce neuronal excitotoxicity, the present study tests this hypothesis in the CNS. Here, we report that intraperitoneal injection of amikacin results in significant damage to cochlear structures [27, 28]. Neuronal damage caused by various injuries, including excitotoxicity, is associated with reactive astrocyte and microglia proliferation and macrophage infiltration at the site of injury [26–30].

Memantine, a drug recently approved for the treatment of moderate to severe Alzheimer’s disease [31–37], reduces excitability between hair cells and afferent fibers of the auditory nerve by blocking NMDA receptors. It is thought to inhibit neurotransmission [32, 33]. It probably also acts on the central auditory pathway [36, 38]. Memantine has been previously tested in animals [35, 36] and in humans for the treatment of tinnitus, where it was found to have little or no effect [35–39]. Using an aminoglycoside toxicity model of hair cell and spiral ganglion neuron damage, we show in this report that memantine can protect both cochlear morphology and physiology. Previous studies have shown that NMDA receptors may also contribute to signaling in the mature cochlea [40]. Immunostaining for the GluN1 subunit is robust in adult rats around the base of the IHC [34, 40, 41]. Basel et al. suggested that aminoglycosides cause polyamine-like enhancement of glutamate NMDA receptors, leading to excitotoxicity and eventual hair cell death [41]. These authors showed that NMDA antagonists can prevent hair cell loss and hyperacusis. However, they did not examine neurons in the spiral ganglion, an important relay station between the peripheral auditory organs and the central auditory system. This is particularly important because NMDA receptors are predominantly expressed by neurons in the spiral ganglion rather than hair cells [42, 43].

The results in this report show that NMDA antagonists attenuate spiral ganglion neuron damage, minimizing hair cell loss and positively affecting functional responses. Although some features of the damage caused by noise and aminoglycosides differ, our results suggest that common mechanisms may underlie both. At present, it is unclear whether the protective properties of NMDA antagonists act on hair cells or on spiral ganglion neurons.

The ability of neurotrophic factors to protect spiral ganglion neurons from aminoglycoside toxicity has been previously demonstrated [44]. Neurotrophic factors have been shown to effectively regulate intracellular calcium levels and reduce oxidative damage [45, 46]. These observations suggest that oxidative stress may be a common factor in several auditory pathologies, and additional chemicals have already been reported to reduce the activation of NMDA or its downstream effectors. This reinforces the idea that it is beneficial in protecting the peripheral auditory system from various pathologies, as previously suggested [47–49].

Aminoglycoside antibiotics are widely used in clinical practice and have long been known to cause both cochlear and vestibular ototoxicity. Loss of the outer hair cells of the organ of Corti at the base of the cochlea is the first evidence of aminoglycoside ototoxicity.

As experimental model, Wistar rats have been chosen for the present study. In general, many similarities can be found between the auditory anatomy of the rat, other rodents, and humans. For example, rats have both inner hair cells (IHC) in a single row and outer hair cells (OHC) in three rows arranged along the organ of Corti [50], and these sensory cells are more compressed in the basal turn than in the apical turn of the cochlea [40, 42].

## Conclusion

As a conclusion of the experimental work, it is found that co-administration of memantine with aminoglycos To a lesser extent, it appears that administration of memantine may reduce the aforementioned ototoxicity immediately after discontinuation of amicacin administration. ide (in this case, amicacin) can reduce the ototoxic effect of the latter to a certain extent.

Future studies should further investigate the dose-dependent changes in DPOAE in animals treated with amikacin and memantine and the combined pharmacokinetics of these two drugs and explore the mechanisms of enhanced ototoxicity.

## Data Availability

The data presented in this study are available on request from the corresponding author.

## Data Availability

The data presented in this study are available at reasonable request from the corresponding author. The data are not publicly available due to ethical/privacy restrictions.
